# MODIFIABLE FACTORS IN COGNITIVE HEALTH OF THE RURAL AGING POPULATION: A SCOPING REVIEW

**DOI:** 10.1093/geroni/igae098.0777

**Published:** 2024-12-31

**Authors:** Ladan Ghazi Saidi, Vaishali Phatak, Miechelle McKelvey

**Affiliations:** University of Nebraska Kearney, Kearney, Nebraska, United States; University of Nebraska Medical Center, Omaha, Nebraska, United States; University of Nebraska Kearney, Kearney, Nebraska, United States

## Abstract

**Introduction:**

This presentation highlights modifiable risk factors that impact the cognitive well-being of older adults in rural regions. Studies on rural population are rare due to its challenges. Considering the global distribution of the older adults, with more than 20% of America’s older population residing in rural locales, it is essential to recognize the specific risk factors inherent to these areas to foster healthy aging. We summarize the literature on cognitive health at aging in rural populations.

**Method:**

Articles were sourced from multiple databases and were subjected to rigorous inclusion and exclusion criteria following PRISMA guidelines. The initial pool of 92 articles was refined to 13 for an in-depth analysis.

**Findings:**

The findings from our scoping review reveal three primary elements related to the rural context affecting cognitive health. We conclude that disparities are associated with demographics and healthcare access; facets of quality of life like physical activity, sleep patterns, and social engagement are essential in healthy aging; and overall physical and mental health play a vital role in healthy cognition in aging.

**Conclusion:**

Research on healthy aging and Alzheimer’s Disease and Related Dementias (ADRD) should consider rural residency as a possible risk factor.

